# Mitochondrial fitness sustains healthy muscle aging

**DOI:** 10.18632/aging.204857

**Published:** 2023-06-24

**Authors:** Andrea Irazoki, Antonio Zorzano, David Sebastián

**Affiliations:** 1 Department of Biomedical Sciences (BMI), University of Copenhagen, Copenhagen, Denmark; 2 Institute for Research in Biomedicine (IRB Barcelona), The Barcelona Institute of Science and Technology, Barcelona,Spain; 3 Departament de Bioquímica i Biomedicina Molecular, Facultat de Biologia, Universitat de Barcelona, Barcelona, Spain; 4 Centro de Investigación Biomédica en Red de Diabetes y Enfermedades Metabólicas Asociadas (CIBERDEM), Instituto de Salud Carlos III, Madrid, Spain; 5 Department of Biochemistry and Physiology, School of Pharmacy and Food Sciences, Universitat de Barcelona, Barcelona,Spain

**Keywords:** mitochondria, mitophagy, mitochondrial dynamics, aging, sarcopenia

Aging is a biological process associated with a time-dependent functional decline leading to tissue dysfunction and failure. In the context of skeletal muscle, a key manifestation of aging is the progressive loss of muscle mass and function, also referred to sarcopenia. Sarcopenia is a fundamental contributor to disability and loss of autonomy in the elderly, leading to a decrease in the quality of life [1]. Skeletal muscle alterations also play a central role in the development of age-associated metabolic disease. Therefore, the understanding of the molecular determinants of age-induced alterations in skeletal muscle is of great interest to promote healthy aging.

A hallmark of muscle aging is the buildup of dysfunctional and damaged mitochondria. However, the mechanisms leading to the accumulation of unhealthy mitochondria and whether this drives some of the aging-induced alterations are not fully understood yet. The process responsible for the selective degradation of damaged mitochondria, also known as mitophagy, is key in the maintenance of mitochondrial quality. Besides, mitophagy is tightly tuned with mitochondrial dynamics, and this coordination is essential during mitochondrial quality control [2, 3]. Indeed, alterations in these processes have been found to contribute to the accumulation of dysfunctional mitochondria in aged muscles [4, 5]. In particular, we have shown that a reduction in the mitochondrial fusion protein Mitofusin 2 (Mfn2) during aging drives metabolic deterioration, muscle atrophy and sarcopenia by a deregulation of mitochondrial dynamics and mitophagy [4]. Interestingly, as a consequence of the accumulation of damaged and ROS-generating mitochondria, an adaptive mitophagy pathway involving ROS-induced expression of the mitophagy protein BNIP3 is activated in order to minimize mitochondrial damage. Pharmacological inhibition of this adaptive mitophagy pathway [4, 6] or genetic downregulation of muscle BNIP3 [6] worsens mitochondrial quality and potentiates muscle atrophy. In contrast, re-expression of Mfn2 to levels comparable to those of young mice prevents muscle atrophy in old mice [4]. Altogether, these data demonstrate a tight connection between mitochondrial health and the development of muscle atrophy and sarcopenia.

Damaged mitochondria also contribute to the triggering of inflammatory responses by the release of mitochondrial damage-associated molecular patterns (mtDAMPs) [7]. Therefore, the accumulation of unhealthy mitochondria potentially drives the induction of inflammation. Importantly, the presence of chronic inflammation is a contributing factor for the development of sarcopenia [8]. However, whether loss of muscle mitochondrial fitness could orchestrate the induction of inflammation and sarcopenia in the context of aging had not been explored to date. In a recent study, we have addressed this riddle by evaluating the role of BNIP3 in controlling mitochondrial health and inflammation during aging in skeletal muscle. In order to obtain a comprehensive view of the molecular and physiological events linking loss of mitochondrial fitness and inflammation, we have used a range of tools, including cultured myotubes, aged mice, and human muscle biopsies from young and aged subjects [6].

Downregulation of BNIP3 protein expression in cultured myotubes disrupts mitophagy and leads to mitochondrial dysfunction. Strikingly, BNIP3 repression also causes lysosomal dysfunction, leading to an accumulation of undigested autolysosomes. This suggests that this protein tunes both mitochondrial and lysosomal function in order to sustain mitophagic activity. Importantly, these alterations caused by reduced levels of BNIP3 lead to an enhanced interaction of mitochondrial DNA (mtDNA) and the DNA sensor Toll-like receptor 9 (TLR9) in lysosomes/autolysosomes, leading to an upregulation of inflammatory genes and increased secretion of pro-inflammatory cytokines. In mice, aging is associated to an increase in BNIP3 protein expression in muscle, and consistent with the *in vitro* data, downregulation of BNIP3 in muscle from aged mice aggravates inflammation and muscle atrophy, characterized by decreased cross-sectional area (CSA), upregulated atrophy-related gene/atrogene expression, and increased denervation. Lastly, to provide further physiological support to these findings, we analyzed BNIP3 expression and muscle health status in human muscle biopsies from young and aged subjects. BNIP3 expression is increased in aged subjects, suggesting that age-induced BNIP3 modulation is conserved in mice and humans. Importantly, when stratifying aged subjects according to low and high BNIP3 expression in muscle, we detected a tight association between BNIP3 protein levels, inflammation and the probability of developing sarcopenia. These data suggest that the age-induced increase in BNIP3 protein levels in muscle constitutes a protective mechanism in a context of a general decrease in mitophagy, which mitigates mitochondrial damage and confers resistance to age-induced inflammation and muscle atrophy. Overall, our data reveal a new molecular mechanism by which loss of mitochondrial fitness leads to inflammation and unhealthy muscle aging.

**Figure 1:**
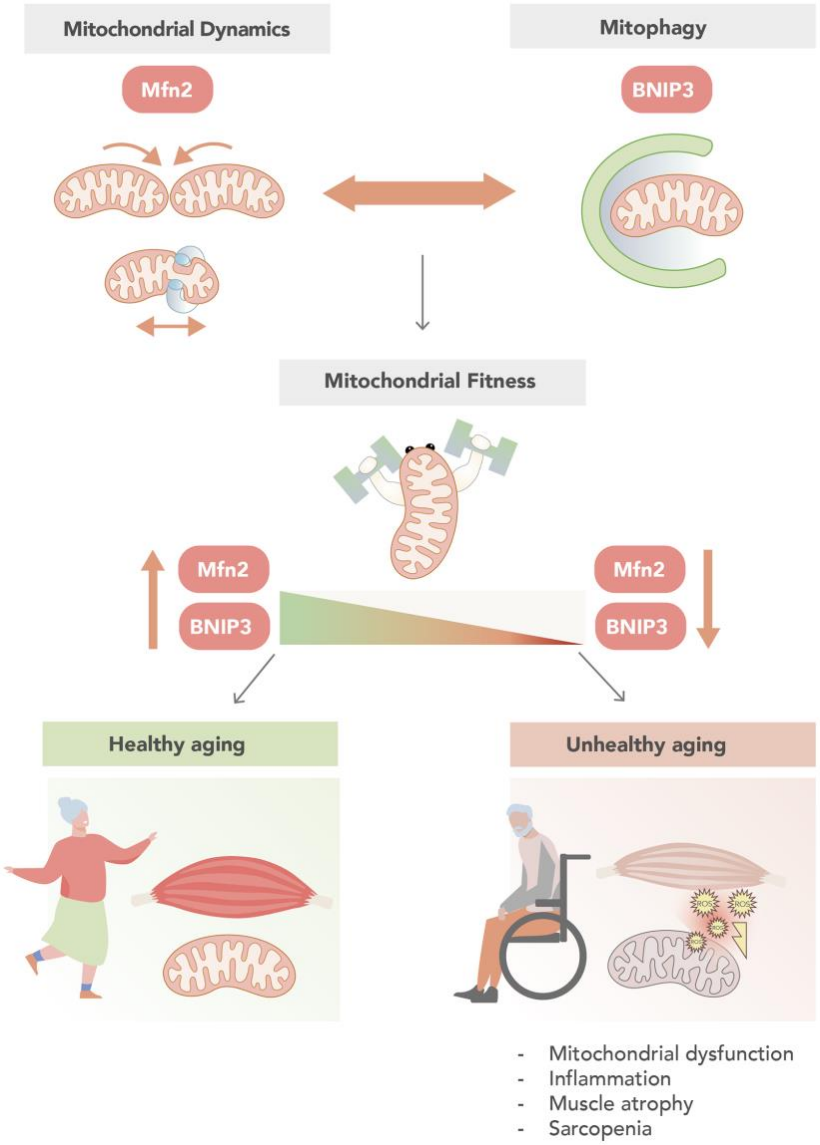
**Mitochondrial fitness sustains healthy muscle aging.** Coordination of mitochondrial dynamics and mitophagy are two key events in the control of mitochondrial fitness. In this regard, high levels of the mitochondrial dynamics protein Mfn2, and the mitophagy protein BNIP3, are associated with an increase in mitochondrial fitness and determine a healthy muscle aging. In contrast, unhealthy muscle aging is associated to low levels of Mfn2 and BNIP3, which is characterized by mitochondrial dysfunction, inflammation, muscle atrophy and sarcopenia.

Based on these findings, we propose that alterations in mitochondrial dynamics and mitophagy lead to the loss of mitochondrial fitness observed during aging, and they constitute a pivotal factor leading to age-related alterations, such as inflammation, metabolic disturbances, and the development of sarcopenia (Figure 1). Evidently, many questions remain to be addressed, such as how altered mitochondrial dynamics affect mitophagy, how reduced BNIP3 expression causes an inflammatory response in cells and in muscle, and whether inflammation *per se* is a determinant of unhealthy aging. In addition, other mitochondrial quality control mechanisms, such as mitochondrial biogenesis and mitochondrial unfolded protein response (mtUPR) could also have a role in the accumulation of damaged mitochondria during aging. Therefore, future studies should focus on the discovery of the precise factors -whether they are genetic or environmental-leading to decreased mitochondrial health during aging, and whether they can be modulated. These studies represent an untapped opportunity for medical exploitation aiming to promote healthy aging.
